# Study on the Occurrence Characteristics and Control Techniques of Gummosis in *Zanthoxylum bungeanum* on the Qinghai Plateau

**DOI:** 10.3390/jof11120860

**Published:** 2025-12-03

**Authors:** Junlong Feng, Shuqun Deng, Dong Zhao, Chenxu Gao, Yan Han, Yang Zhang, Hongyu Chen, Wei Li

**Affiliations:** 1 Academy of Agricultural and Forestry Sciences, Qinghai University, Xining 810016, China; 18292334028@163.com (J.F.); ds360265@163.com (S.D.); zd18028@163.com (D.Z.); gcx13700588191@163.com (C.G.); 13709718399@163.com (Y.H.); qhzhangyang2025@163.com (Y.Z.); 2 Key Laboratory of Integrated Management of Agricultural Pest in Qinghai Province, Xining 810016, China; 3 Analysis and Testing Center, Qinghai Academy of Agricultural and Forestry Sciences, Xining 810016, China

**Keywords:** gummosis of *Zanthoxylum bungeanum*, *Fusarium equiseti*, ethylicin, disease quantification, cellular ultrastructure

## Abstract

As an important economic forest tree species on the Qinghai Plateau, the outbreak of gummosis in *Zanthoxylum bungeanum* seriously threatens the sustainable development of the regional industry. This study is the first to clearly identify *Fusarium equiseti* as the pathogen responsible for the gummosis disease of *Z. bungeanum* on the Qinghai Plateau. To identify effective control agents, based on the indoor virulence screening of 13 fungicides, it was found that 80% ethylicin exhibited the strongest inhibitory activity against the pathogen (EC_50_ = 0.396 μg/mL). Transmission electron microscopy revealed that ethylicin acts through multi-target effects, including disruption of cell membrane integrity and induction of mitochondrial cristae disintegration. Field trials have shown that the control efficacy of 80% ethylicin alone reaches 93.10%, and when combined with rhamnolipid in a 1:1 ratio, the control efficacy remains at 85.77% even when its dosage is reduced by 50%. Additionally, a novel Xuan paper imprint method was developed for precise quantification of lesion areas in the field. This study demonstrates the potential of ethylicin as a core fungicide for controlling *Z. bungeanum* gummosis, offering a scientific basis for integrated disease management in plateau ecosystems.

## 1. Introduction

As a member of the Rutaceae family, *Zanthoxylum bungeanum* is an economically significant tree species. Its pericarp contains abundant volatile components with unique value in food processing. *Z. bungeanum*, under the unique plateau climate, low-temperature conditions, and nutrient-rich soil environment of the Qinghai Plateau [1,2,3], exhibits higher amide compound content and superior overall quality compared to other regions [4]. However, in recent years, the outbreak of gummosis has led to the decline in tree vigor, significant reduction in fruit yield and quality, severely constraining the sustainable development of the local *Z. bungeanum* industry.

The typical characteristic of *Z. bungeanum* gummosis is the secretion of yellowish-brown to amber gum by infected tissues, leading to tree weakness and even death, which is primarily caused by fungal pathogens. Recent studies indicate that *Fusarium* spp. are a key pathogenic group responsible for gummosis in *Z. bungeanum*, with significant species diversity. For instance, in the Longnan region of Gansu Province, *Fusarium tricinctum* has been identified as a major pathogen, often co-infecting with *Dothiorella* spp. [5]. In Ya’an, Sichuan Province, *Fusarium fujikuroi* has been reported to cause black stem rot accompanied by gummosis symptoms [6], while in Hanyuan, the pathogens were identified as *Fusarium tricinctum* and *Phomopsis perniciosa* [7]. Additionally, *Phytophthora* spp., such as *P. citrophthora*, have also been reported as pathogens in certain areas [8]. These findings collectively reveal the diversity and complexity of fungal pathogens causing gummosis across different *Z. bungeanum* production regions in China. However, in the unique alpine and arid environmental conditions of the Qinghai Plateau, the specific fungal pathogens causing gummosis in *Z. bungeanum* have not yet been clearly identified. Therefore, it is imperative to conduct localized pathogen identification in the Qinghai Plateau to clarify the pathogen composition, thereby laying the foundation for developing efficient and precise control strategies.

The current control of gummosis primarily relies on chemical and biological methods. The efficacy of chemical control depends on the selection of agents, the method of application, and the mechanism of action. Studies have shown that spraying benomyl after pruning can effectively prevent pathogen infection [9,10]; applying 10% polyoxin wettable powder using the “scraping and coating method” at the early stage of lesion development can reduce gummosis by 59.38% [11]. The compound formulation study showed that the corrected efficacy of the mixture of lime sulfur and carbendazim reached 72.4% [12]; the relative efficacy of the combination of 1.5% polyoxin wettable powder and 30.0 mg/L 2,4–D could exceed 96% [13]. Biological control is primarily achieved through antagonistic microorganisms. *Trichoderma harzianum* significantly reduces the incidence of peach tree gummosis by producing volatile organic compounds, hyphal entanglement, and degradation of hyphae of the pathogen [14]. However, currently in China, the only registered pesticide for controlling gummosis is the microbial preparation of *Paenibacillus polymyxa*, and there is a lack of registered chemical pesticides. Therefore, to meet the needs of crops in the Qinghai Plateau region, it is urgent to identify a safe and reliable pesticide agent. Ethylicin is an organosulfur plant-bionic pesticide independently developed in China, and is the ethyl homolog of allicin [15]. The active groups in the molecular structure of this agent can specifically bind to sulfur-containing metabolic enzymes in pathogenic fungi, thereby effectively controlling a variety of fungal diseases. It is characterized by broad-spectrum efficacy, low toxicity, rapid action, and environment friendly. Studies have shown that ethylicin can significantly inhibit the mycelial growth, sporulation, and spore germination of fungi. Its mechanism of action involves interfering with the protein synthesis of pathogens by inhibiting ribosome function and amino acid metabolism [16]. The combined use of ethylicin with other fungicides can effectively control potato late blight and increase potato yield [17].

At the same time, in order to enhance the control efficacy and utilization efficiency of ethylicin, reduce its application rate, and mitigate potential environmental impacts, this study introduced rhamnolipid as a synergist. Rhamnolipid is a biosurfactant produced by microbial metabolism [18], characterized by its ability to reduce surface tension of solutions, enhance wettability and permeability, and disrupt microbial cell membranes [19]. When combined with ethylicin, it is expected to enhance the efficacy through synergistic effects and achieve reduced application of pesticides.

Based on the potential antimicrobial activity of ethylicin against pathogens such as *Fusarium* spp., and the synergistic potential of rhamnolipids [20], this study aims to elucidate the occurrence characteristics of gummosis in *Z. bungeanum* on the Qinghai Plateau and identify its pathogenic fungi, thereby verifying the efficacy of ethylicin and its combination with rhamnolipids in controlling this disease and developing relevant gummosis detection technologies. By exploring effective chemical control strategies, this research provides scientific basis and technical support for the prevention and control of gummosis in *Z. bungeanum* on the Qinghai Plateau, pesticide registration, and the sustainable development of the *Z. bungeanum* industry.

## 2. Materials and Methods

### 2.1. Isolation and Identification of Pathogens

#### 2.1.1. Sample Collection

**Figure 1:**
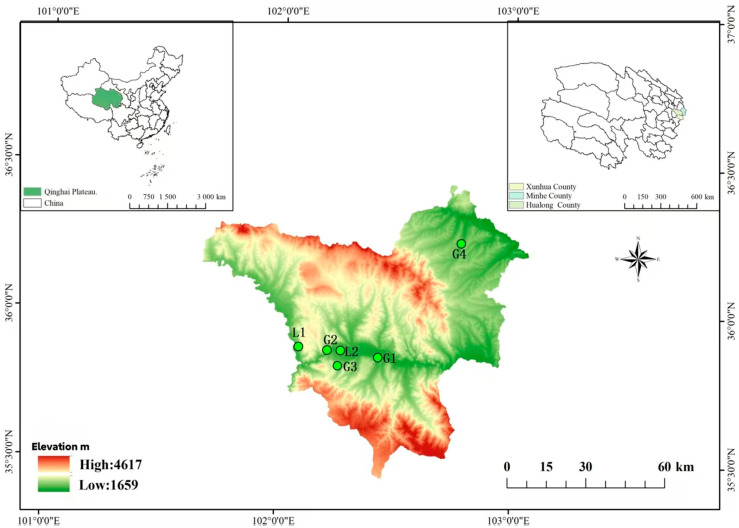
The main *Z. bungeanum* planting areas in Qinghai Plateau. Where the sampling points in the figure are marked with green dots, and geographic coordinates are provided for each point. G1: 102.444059 °E, 35.877288 °N; G2: 102.254401 °E, 35.875697 °N; G3: 102.27356 °E, 35.862198 °N; G4: 102.75047 °E, 36.253766 °N; L1:102.087186 °E, 35.891349 °N; L2:102.269846 °E, 35.875368 °N.

During the 2024 outbreak period of *Z. bungeanum* gummosis, samples exhibiting typical symptoms of gum disease were collected from six representative *Z. bungeanum* planting areas in three major production counties: Xunhua Salar Autonomous County, Minhe Hui and Tu Autonomous County, and Hualong Hui Autonomous County, Qinghai Plateau (Figure 1). Using a sterile scalpel, tissue blocks of 5 × 5 mm were excised at the junction of diseased and healthy tissues. After surface disinfection with 75% ethanol (Sinopharm Chemical Reagent, Shanghai, China) (10 s) [21], the samples were rinsed three times with sterile water and placed in modified Martin’s agar medium (Qingdao Hope Bio-Technology, Qingdao, China) (yeast extract 2.0 g/L, glucose 20.0 g/L, peptone 5.0 g/L, pH 6.8 ± 0.2) for culture grown in the dark at 25 °C for 5 days. When microbial colonies emerge on the surface or edges of plant tissue segments placed on culture medium, single colonies differing in location, size, and morphology are picked and inoculated into a new culture medium using the parallel streak method, and incubated at 28 °C. After new colonies have grown, colonies are selected again for purification based on differences in colony color and morphology. This purification process is repeated until a single strain is isolated.

Concurrently with the collection of these symptomatic branch samples (described above for pathogen isolation), rhizosphere soil samples (0–20 cm depth) were also collected from the base of the same diseased *Zanthoxylum* trees in the three main production areas of Xunhua County, Minhe County, and Hualong County in Qinghai Province. Three independent samples were collected from each planting area, transported in ice boxes to a professional sequencing institution (Shaanxi Aiyouji Biotechnology Co., Ltd., Yangling, China) for sequencing. The total DNA from soil was extracted using the CTAB method, and PCR amplification was performed using fungal ITS1–ITS2 region-specific primers (*ITS1F*: 5′–CTTGGTCATTTAGAGGAAGTAA–3′; *ITS2R*: 5′–GCTGCGTTCTTCATCGATGC–3′). After purification, PE250 paired-end sequencing was conducted on the Illumina NovaSeXq 6000 platform (Illumina, Inc., San Diego, CA, USA). After quality control, the raw sequences were annotated for species. The relative abundance of fungal taxa at the phylum, genus, and species levels was calculated to directly compare the fungal community composition between the rhizosphere soil of diseased plants and that of healthy controls, thereby analyzing the distribution characteristics of core pathogenic fungi.

#### 2.1.2. Morphological and Molecular Identification

The purified strain was stained with lactophenol cotton blue and observed under an optical microscope (40×) for spore morphology and sporulation structures. Genomic DNA was extracted using the CTAB method [22], and PCR amplification was performed with fungal universal primers *ITS*, *TEF–1α*, *LSU rRNA*, and *β–tubulin* (Table 1). The amplified products were detected by 1.2% agarose gel electrophoresis and then sent to Shaanxi Aiyouji Biotechnology Co., Ltd. for sequencing.

The obtained sequences were assembled and aligned using multi-gene assembly and then compared with the NCBI database via BLAST (version 2.15.0, National Center for Biotechnology Information, Bethesda, MD, USA) to identify sequences with a similarity greater than 98%. A phylogenetic tree was constructed using the Neighbor-Joining (NJ) method with MEGA 7.0 software (bootstrap = 1000). The final assembled gene sequences were uploaded to the NCBI database to obtain accession numbers (Table 2).

#### 2.1.3. Pathogenicity Verification of Isolated Fungi

For each isolate, 10 healthy *Z. bungeanum* trees (3-year-old ‘Dahongpao’ cultivar) were selected. The bark surface of the trunk was rinsed thoroughly with sterile water, then disinfected with a cotton ball soaked in 75% ethanol and dried with sterile absorbent paper. Three wounds (5 mm in diameter) were created per tree at 10.0 cm intervals on the main trunk. A mycelial plug (0.5 cm in diameter) obtained from the edge of a colony grown on modified Martin’s agar medium was placed onto each incision with the mycelial side facing downward. The inoculated area was wrapped with moist sterile absorbent paper and covered with sterile aluminum foil. Disease progression was monitored weekly. Re-isolation was performed using conventional tissue separation methods, and the re-isolated strains were incubated at 25 °C for 5 days in a constant-temperature incubator. Colony morphology was compared to that of the original inoculated strains to confirm pathogen identity.

### 2.2. Fungicides Compound Resistance Plate Assay

This experiment used the highly viable strain G of *F. equiseti*, verified by Koch’s postulates, for the virulence assay. To comprehensively screen for effective control agents, we selected 13 commonly used agricultural fungicides. This selection was based on their known efficacy against gummosis in other tree species and their diverse mechanisms of action (including quinone outside inhibitors and demethylation inhibitors). Based on preliminary results, each agent was tested at five concentrations (active ingredient content, unit: μg/mL, Table 3). Each concentration was tested with three replicate plates (biological replicates), and the experiment was conducted twice independently to ensure reproducibility. The specific procedures were as follows: the fungicide-containing medium was evenly poured into Petri dishes (9 cm), inoculated with a 5-day-old fungal plug, and incubated at 25 °C for 5 days [23]. The colony diameter was measured using the cross method, and the inhibition rate of mycelial growth was calculated. The concentration–response data were then subjected to toxicity regression analysis to determine the median effective concentration (EC_50_) for each fungicide. The relative inhibitory activity of the fungicides was evaluated based on their EC_50_ values.

### 2.3. Field Efficacy Trials

#### 2.3.1. Experimental Design

The field experiment was conducted in a severely affected area of Xunhua County, utilizing 3-year-old ‘Dahongpao’ *Z. bungeanum* trees planted at 3.0 m × 4.0 m spacing. The orchard had a pre-existing gummosis incidence of 40–50% and no history of specific chemical control for this disease. Four treatments were established in a randomized block design with three replicates: T1: 80% ethylicin single agent, T2: ethylicin–rhamnolipids 1:1 combination, T3: ethylicin–rhamnolipids 1:3 combination (*v*/*v*), and CK: water control. Each plot contained 50 plants, all of which were monitored for disease progression. Treatment was administered using a brush application method (solution amount: 2.0 mL/cm^2^) once at the onset of the disease (May 25, when approximately 5% of trees showed initial gummosis symptoms) and again 30 days later.

#### 2.3.2. A New Method for Quantitative Determination of Gummosis Lesions

**Figure 2:**
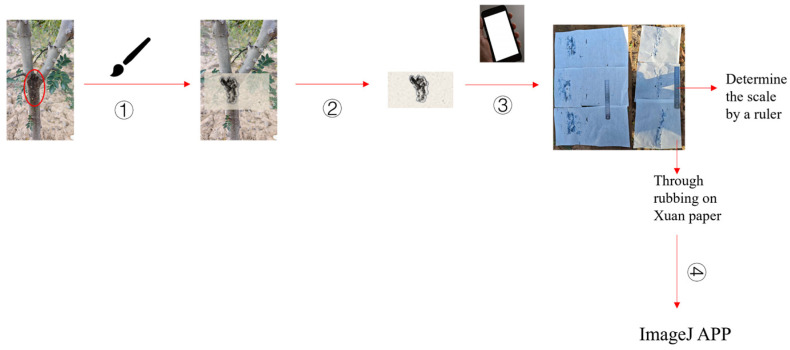
Investigation method of disease spot area of single tree. Note: ① Apply ink to the lesion using a brush. ② Imprint method with Xuan paper. ③ Take a photo with a mobile phone and determine the scale bar. ④ Upload the photo to Image software to measure the area. The red circles indicate the gummosis lesions.

In this study, a self-developed Xuan paper imprint method was employed for precise measurement of lesion areas: first, a brush was used to evenly apply ink to the surface of the target lesion, followed by covering the lesion area with semi-raw Xuan paper and gently pressing for 30 s to ensure a complete rubbing. After the rubbing sample was naturally air-dried, a mobile phone was used to take photographs to obtain digital images. Finally, the ImageJ 1.53 image analysis software was utilized to measure the pixel values of the actual lesion area (S) and the total area of the Xuan paper (S_0_) (Figure 2). Establish a grading standard for gummosis, divided into 6 levels: Level 0 (0%, no lesions) represents healthy plants; Level I (<10%) indicates the initial stage of localized mild infection; Level II (10–20%) reflects moderate spread; Level III (20–30%) shows that the lesions have formed a ring structure; Level IV (30–40%) indicates damage to the vascular tissue; and Level V (>40%) signifies severe epidermal cracking and restricted growth in plants. This grading system achieves digital expression of disease severity through representative values (0–5). The disease incidence per plant and the disease index were calculated according to the formulas described in Section 2.5.3. The reliability of the Xuan paper imprint method was validated using ten randomly selected diseased trees. To ensure comprehensive sampling, all gummosis lesions present on each tree were systematically included in the assessment. Three replicate impressions were obtained from each lesion. All field imprinting and subsequent digital analyses were performed by a single trained operator to maintain procedural consistency throughout the data collection. The consistency of these replicate measurements was quantitatively assessed using the intraclass correlation coefficient (*ICC*) and the coefficient of variation (*CV*).

### 2.4. SEM Analysis of Fungal Hyphae Morphology

SEM observation: Hyphal samples of *F. equiseti* (strain G) were harvested from 5-day-old colony edges on modified Martin’s agar using a sterile scalpel, then suspended in sterile PBS (pH 7.4). After brief vortexing to remove residual agar, samples were centrifuged (3000 rpm, 5 min) and supernatant discarded. Pellets were fixed in 2.5% glutaraldehyde/0.1 M PBS (4 °C, 4 h), dehydrated in graded ethanol (50%/70%/100%), and critical-point-dried. Samples were mounted on conductive tape, sputter-coated with Au-Pd, and imaged under high vacuum. Ten random fields per sample (three biological replicates) were examined for morphological analysis.

TEM observation: Hyphal samples were processed identically to SEM through fixation. After glutaraldehyde fixation, samples were dehydrated in graded acetone (50%/70%/100%) for improved ultrastructure preservation, then infiltrated with epoxy resin, embedded, and sectioned (70 nm). Ultrathin sections were stained with uranyl acetate and lead citrate prior to TEM observation. Subcellular alterations were analyzed in three biological replicates per treatment.

### 2.5. Statistical Analysis

#### 2.5.1. Experimental Unit Design

In vitro virulence assays: Individual Petri dish (three replicates per concentration; two independent runs). Field efficacy trials: Single *Z. bungeanum* tree (50 trees per plot; three replicate plots). Lesion area quantification: Individual gummosis lesion with three replicate imprints (ten diseased trees sampled).

#### 2.5.2. Statistical Analysis Methods

EC_50_ determination: Probit analysis was conducted by modeling the concentration–response relationship through linear regression, using log_10_-transformed fungicide concentrations and probit-converted inhibition rates. Comparative analysis: One-way analysis of variance (ANOVA) was applied to both inhibition rates and field efficacy data. The field trial data for ethylicin and rhamnolipid were presented as the mean ± standard deviation of three replicates. Percentage data underwent arcsine transformation prior to ANOVA to meet the assumptions of parametric tests. For cases where ANOVA indicated significant differences (*α* = 0.05), Tukey’s honestly significant difference (HSD) test was employed for post hoc multiple comparisons. Reliability assessment: The consistency of lesion area measurements from repeated Xuan paper imprints was evaluated by calculating the intraclass correlation coefficient under a one-way random-effects model [*ICC* (1,1)].

#### 2.5.3. Key Calculation Formula

Disease incidence per plant (*PDPI*):

$$\begin{array}{c} \mathrm{PDPI}\left(\%\right)=\frac{\sum_{i=1}^{n}S_{i}}{\sum_{i=1}^{n}S_{0i}}\times 100 \end{array}$$

where *Sᵢ* is the actual lesion area (cm^2^) on the i-th sampled plant (measured via Xuan paper imprint), and *S_*0*_ᵢ* is the total area (cm^2^) of the Xuan paper used for imprinting the lesion on the i-th plant.

Disease index (*DI*):

$$DI\left(\%\right)=\frac{\sum_{i=1}^{n}\left(Number\;of\;diseased\;plants\times This\;level\;represents\;the\;numerical\;value\right)}{Total\;number\;of\;plants\;surveyed\times 5}$$

where n is the disease grade (0–5).

Inhibition rate of mycelial growth (%):

$$\mathrm{Inhibition}\;\mathrm{rate}\;(\%)\;=\;\begin{array}{c} \left(\frac{Dc-Dt}{Dc-5}\right)\times 100\; \end{array}$$

where *Dc* is the average diameter (mm) of the control colony, and *Dt* is the average diameter (mm) of the treated colony.

Virulence regression equation:

y=a+bx

where *x* is the logarithm of the fungicide concentration (log_10_ μg/mL), and y is the probit value of the inhibition rate.

Intraclass Correlation Coefficient:

$$ICC_{\left(1,1\right)}=\frac{MS_{B}-MS_{W}}{MS_{B}+\left(k-1\right)\cdot MS_{W}}$$

Coefficient of Variation: $\begin{array}{c} CV\left(\%\right)=\frac{\sigma }{\mu }\times 100 \end{array}$

$$95\%\;\mathrm{Confidence}\;\mathrm{Interval}:\;ICC_{\mathrm{lower}}=\frac{F/F_{\mathrm{upper}}-1}{F/F_{\mathrm{upper}}+k-1},ICC_{\mathrm{upper}}=\frac{F/F_{\mathrm{lower}}-1}{F/F_{\mathrm{lower}}+k-1}$$

where *MS*_B_ denotes the mean square between groups. *MS*W denotes the mean square within groups. k represents the number of repeated measurements per sample (*k* = 3 in this study). An *ICC* value closer to 1 indicates better repeatability. σ denotes the standard deviation. μ denotes the mean. A smaller *CV* value indicates higher data stability. *F* denotes the variance ratio (*F* = *MS*_B_/*MS*_W_) following an *F* distribution. Fupper denotes the upper critical value of the F distribution at the *α*/2 significance level. Flower denotes the lower critical value of the *F* distribution at the *α*/2 significance level (*α* = 0.05 for 95% confidence intervals in this study).

#### 2.5.4. Analysis Tools and Software

Probit regression and EC_50_ calculations were performed in Origin Pro 2022 (version 2022, OriginLab Corporation, Northampton, MA, USA). Phylogenetic analysis and tree construction used MEGA 7.0. (version 7.0, Penn State University, University Park, PA, USA) Parametric tests (ANOVA, Tukey’s HSD, *ICC*) were executed in SPSS 26.0. (version 26.0, IBM Corporation, Armonk, NY, USA) Lesion area quantification employed ImageJ 1.53 (version 1.53, National Institutes of Health, Bethesda, MD, USA) for pixel-based measurements.

## 3. Results

### 3.1. Field Disease Sample Collection and Disease Incidence Patterns

**Figure 3:**
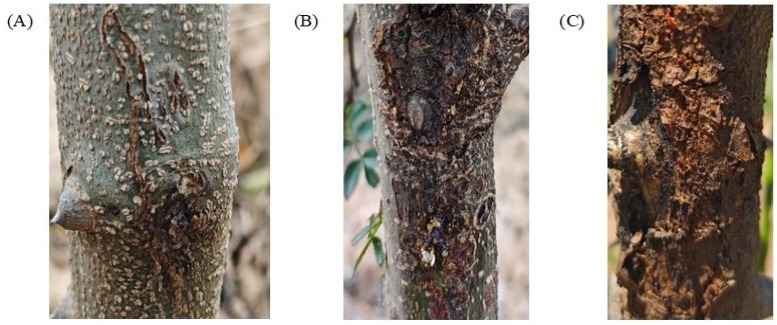
Field symptoms of *Z. bungeanum* tree gummosis. Where (**A**): early stage of the disease, (**B**): intermediate stage of the disease, (**C**): advanced stage of the disease.

The occurrence of gummosis in *Z. bungeanum* trees in Xunhua, Qinghai, is closely related to climate and tree conditions. The initial onset typically begins in mid to late May, as temperatures rise above 15 °C, activating the pathogenic fungi. During the rainy season from July to August, the high temperature and humidity accelerate the spread of the fungi, significantly worsening the condition, especially in orchards with clay-rich, compacted soil exhibiting poor drainage. The peak period of the disease occurs from late August to September, when concentrated rainfall and suitable temperatures facilitate rapid fungi reproduction, leading to widespread gummosis exudation on tree trunks. In the early stages, the disease manifests as reddish-brown sunken spots at the base or on the bark of branches, exuding yellow transparent gummosis. In the mid-stage, the spots expand into irregular shapes, with the gummosis turning from yellow to brown, accompanied by bark rot and discoloration of the xylem. In severe cases, the spots dry, shrink, and crack, causing leaves to yellow, branches to wither, and even the entire plant to die (Figure 3).

### 3.2. Diversity of Pathogens and Identification of Dominant Species

#### 3.2.1. Root Zone Soil Fungal Community Composition

**Figure 4:**
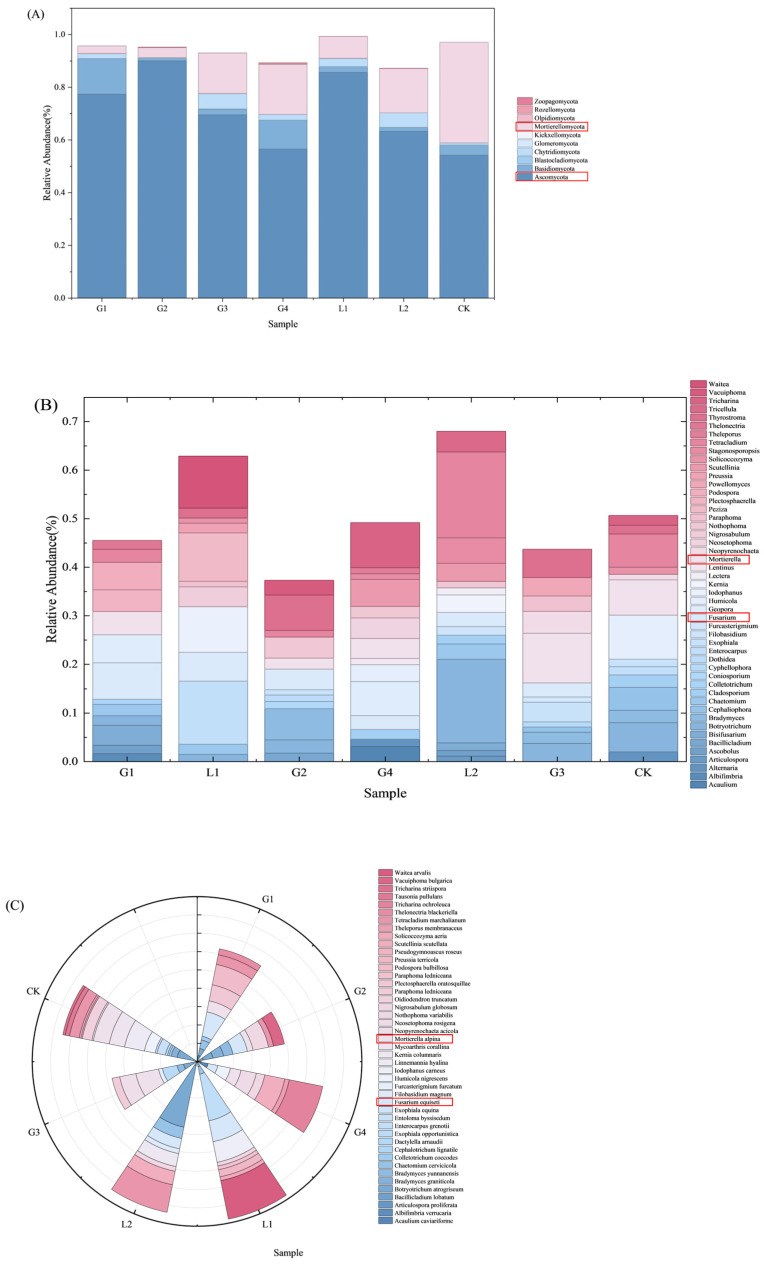
Root zone soil fungal community composition. Where only taxa with a relative abundance ≥1% are annotated. Those highlighted in red represent Mortierellomycota and Ascomycota present at all sampling sites. In the figure, (**A**–**C**) denote species annotations at the phylum, genus, and species levels, respectively.

High-throughput sequencing’s taxonomic analysis provides classification information at various taxonomic levels (Figure 4). By analyzing the relative abundances at levels such as phylum, genus, and species, the composition of (fungal) communities can be determined. In the analysis of fungal community structure, a total of 10 phyla, 49 genera, and 45 species were annotated across all sample groups with a relative abundance greater than 1%. At the phylum level, Ascomycota was the most dominant phylum (relative abundance 54.18–89.98%). The abundance of Ascomycota in the experimental groups was generally higher than that in the control group (CK), with the G2 group (89.98%) being the most significant. The CK group exhibited a unique enrichment of Mortierellomycota (38.19%), which was significantly higher than that in all experimental groups (≤19.04%). The analysis at the genus level revealed that the CK group exhibited the highest richness (14 genera), significantly higher than the experimental groups (with the G3 group being the lowest, containing only 10 genera). The genus *Fusarium* spp. was present in all diseased soils (with an abundance of 2.83–7.45%, highest in the G1 group), but was completely absent in the CK group; at the species level, the richness advantage of the CK group was further confirmed (18 species), while the species richness of the experimental groups significantly decreased (only 7 species in the G3 group).

#### 3.2.2. Isolation and Pathogenicity Verification of Fungi

**Figure 5:**
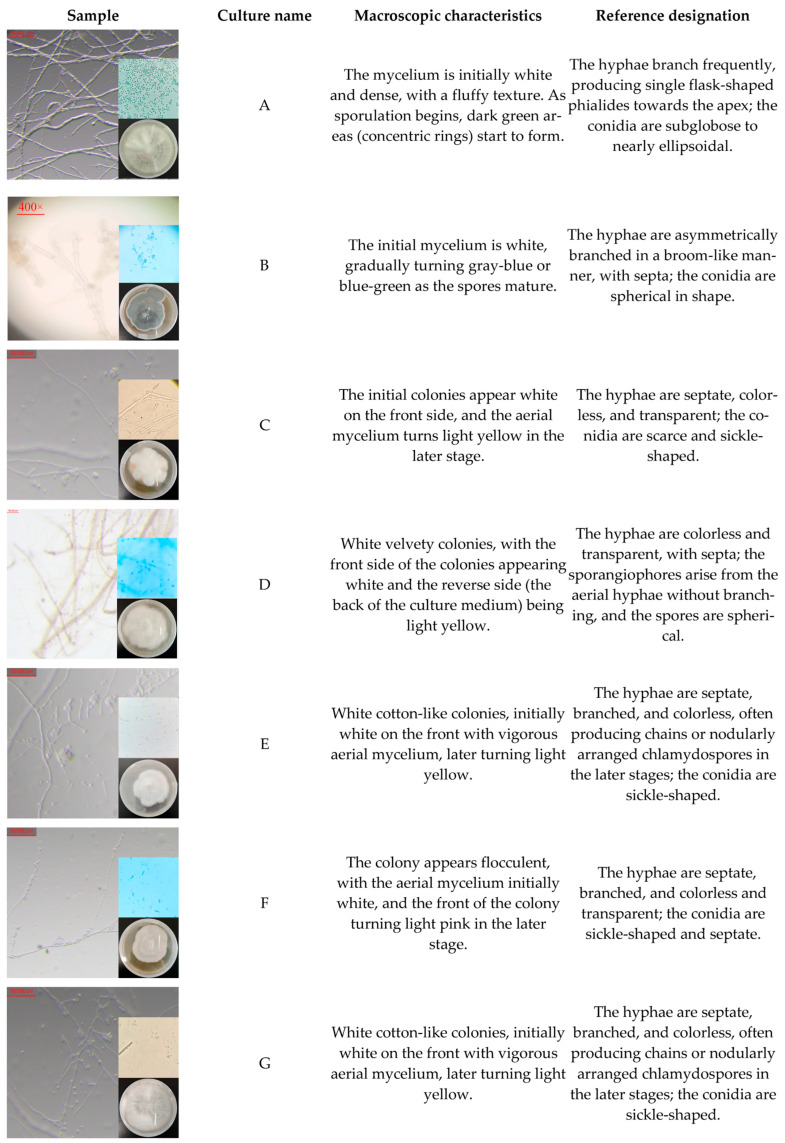
Morphological identification of fungi. Where this image shows a composite of hyphal spores and fungal culture plates.

**Figure 6:**
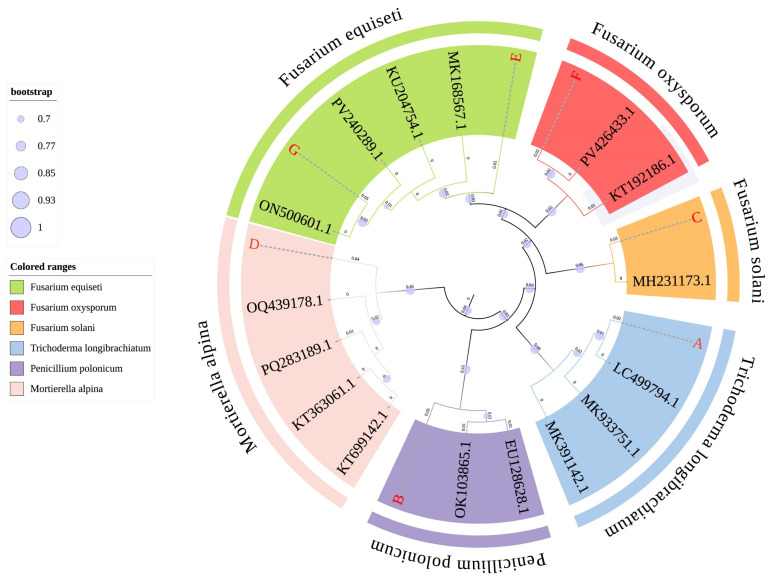
Establishment of a purified fungal developmental tree. Where the multi-gene phylogenetic tree was constructed based on the NJ method (Bootstrap ≥ 70% is shown), with the scale bar indicating the nucleotide substitution rate per site. The strains isolated in this study are highlighted in red. The accession numbers in the figure represent genes with a similarity greater than 98% as determined by BLAST comparison on NCBI.

The seven fungal strains isolated and purified from diseased *Z. bungeanum* stems in Qinghai (isolation procedure detailed in Section 2.1.2) exhibited differences in colony morphology (Figure 5). PCR amplification and sequencing of seven fungal species were performed using two pairs of universal fungal primers. After sequence assembly, a multi-gene phylogenetic tree was constructed by combining the sequences of *TEF–1α*, *LSU rRNA*, and *β–tubulin* genes (Figure 6). The evolutionary tree, built using the Neighbor-Joining (NJ) method shows that the seven fungi isolated and purified from *Z. bungeanum* gummosis disease samples collected from six locations belong to four genera: *Fusarium* spp., *Penicillium* spp., *Trichoderma* spp., and *Mortierella* spp. Through phylogenetic analysis combined with NCBI database comparison, the molecular identification results of the strains are as follows: A—*Trichoderma longibrachiatum*, B—*Penicillium polonicum*, C—*Fusarium solani*, D—*Mortierella alpina*, E—*Fusarium equiseti*, F—*Fusarium oxysporum*, G—*Fusarium equiseti* (E and G are homologous). The *Fusarium* spp. genus is dominant (57.14%), consistent with the composition of core soil pathogens.

After isolating and purifying the tissues, the pathogenicity of the obtained strains was determined. After 14 days of treatment, the areas inoculated with strains E and G appeared water-soaked and blackened, while the *Z. bungeanum* plants inoculated with other strains showed no significant changes. After 45 days of treatment, amber-colored colloids appeared at the inoculation sites of strains E and G, whereas the *Z. bungeanum* trees inoculated with the other strains did not exhibit similar symptoms. No symptoms were observed in the control group inoculated with sterile agar plugs. The severity of disease at the inoculation site was graded according to the criteria in Section 2.3.2: strain E induced Grade I lesions, accounting for 8.24 ± 1.51% (lesion area: 6.59 ± 1.20 cm^2^; paper area: 80.00 cm^2^), strain G induced Grade I lesions accounting for 7.86 ± 1.29% (lesion area: 6.29 ± 1.04 cm^2^; paper area: 80.00 cm^2^), while the other strains exhibited Grade 0 symptoms.

To fulfill Koch’s postulates, infected tissues from symptomatic areas were aseptically excised and subjected to re-isolation using conventional tissue separation methods. The re-isolated strains were incubated at 25 °C for 5 days in a constant-temperature incubator. DNA was extracted from these strains for sequencing, and the obtained sequences were compared with those of the original inoculated strains using MEGA 7.0 software. Phylogenetic analysis confirmed that the re-isolated strains were identical to the inoculated F. equiseti strains (E and G), thereby completing Koch’s postulates and identifying *F. equiseti* as the pathogenic fungus of *Z. bungeanum* gummosis.

### 3.3. Fungicides Resistance Plate Assay

**Figure 7:**
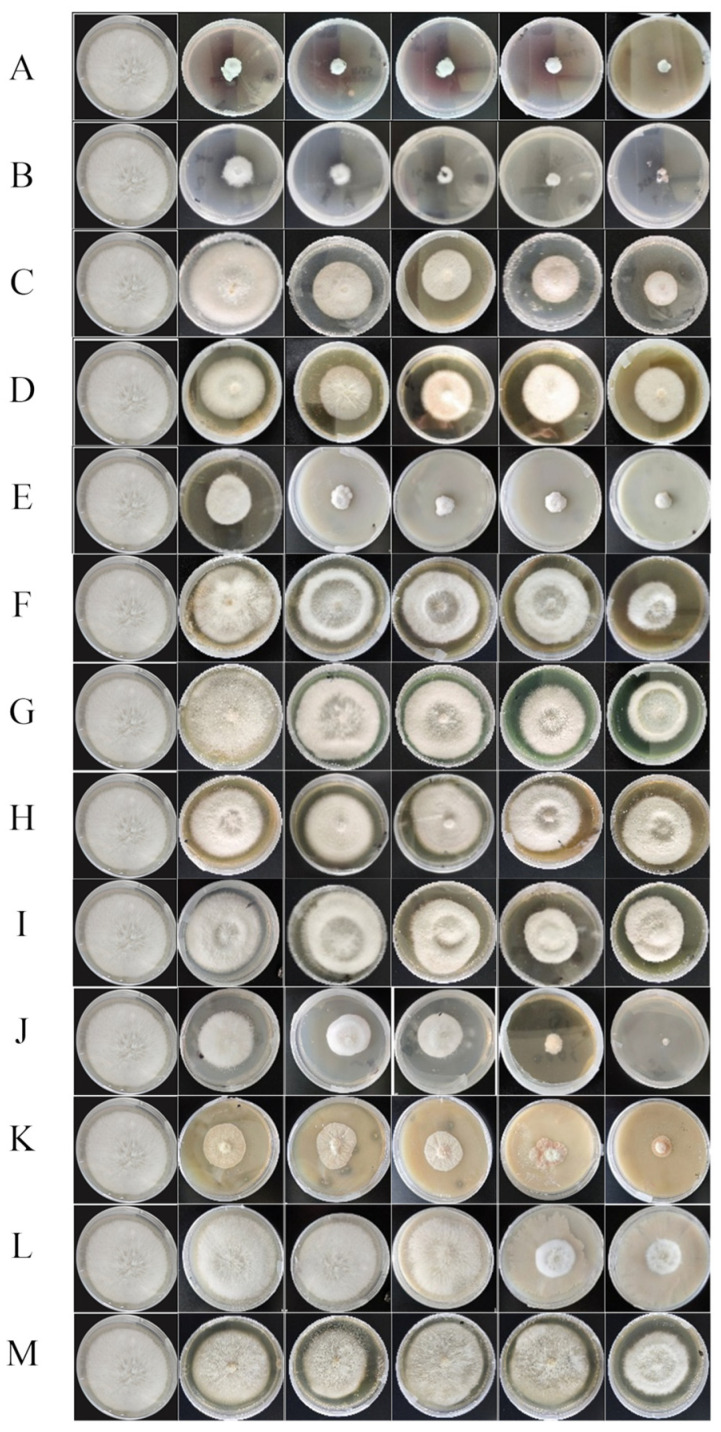
Pesticide inhibition diagram.

Using *F. equiseti* G as the test strain. In this experiment, 13 common fungicides were selected for indoor toxicity testing, with each agent tested at five concentrations (Figure 7). Within the set concentration range, the colony diameter of the tested strains showed a negative correlation with the concentration of the agents, and the inhibition rate significantly increased with concentration. Based on the toxicity regression equations established using the mycelial growth rate method (Table 4). All correlation coefficients (r) exceeded 0.80, confirming strong linear relationships for reliable EC_50_ estimation. Based on their EC_50_ values, the fungicides exhibited a wide spectrum of in vitro efficacy and were categorized into three groups: high (EC_50_ < 1.0 μg/mL), moderate (EC_50_ 1.0–10.0 μg/mL), and low (EC_50_ > 10.0 μg/mL) inhibitory activity. A statistical comparison of relative potency, based on the overlap of 95% confidence intervals, revealed that 80% ethylicin (EC_50_ = 0.396 μg/mL), 90% carbendazim (EC_50_ = 0.447 μg/mL), and 0.3% tetramycin (EC_50_ = 0.464 μg/mL) formed a statistical group with the highest and statistically equivalent inhibitory activity, as their confidence intervals substantially overlapped. Because 80% ethylicin was selected for subsequent field trials and mechanistic studies due to its position within the highest efficacy group, combined with its desirable properties as a plant-bionic and low-toxicity agent.

### 3.4. Field Control Effect and Dose Optimization

#### 3.4.1. A New Method for Quantitative Determination of Gummosis Lesions

To verify the reliability of the Xuan paper imprint method in the quantitative analysis of canker lesions on *Z. bungeanum*, this study randomly selected 10 diseased *Z. bungeanum* trees for methodological evaluation (Table 5). The intraclass correlation coefficient *(ICC*) analysis demonstrated excellent measurement consistency [*ICC* [1,1] = 0.931, 95% *CI* (0.831, 0.977)). Furthermore, the coefficient of variation (*CV*) for all replicate measurements was below 20%, indicating high data stability. These results confirm that the Xuan paper imprint method is a highly reliable and reproducible technique, providing a practical and precise tool for the quantitative assessment of canker disease phenotypes in field conditions.

$$\begin{array}{c} \mathrm{ICC}=\frac{MS_{B}-MS_{W}}{MS_{B}+\left(k-1\right)MS_{W}} \end{array}=\frac{1131.07-27.30}{1131.07+\left(3-1\right)\times 27.30}=\frac{1103.77}{1185.67}=0.931$$

$$ICC_{\mathrm{lower}}=\frac{F/F_{\mathrm{upper}}-1}{F/F_{\mathrm{upper}}+k-1}=\frac{41.4327/2.839-1}{41.4327/2.839+3-1}=\frac{14.592-1}{14.592+2}=\frac{13.592}{16.592}=0.819$$

$$ICC_{\mathrm{upper}}=\frac{F/F_{\mathrm{upper}}-1}{F/F_{\mathrm{upper}}+k-1}=\frac{41.4327/0.270-1}{41.4327/0.270+3-1}=\frac{153.454-1}{153.454+2}=\frac{152.454}{155.454}=0.980$$

#### 3.4.2. Field Effect Test of Ethylicin and Rhamnolipids

To elucidate the synergistic effect of ethylicin and rhamnolipids, this study evaluated the disease index and control efficacy 10 days after the last application. As shown in Table 6, the disease control efficacy of ethylicin alone (1:0), 1:1, and 1:3 combination treatments were 93.10%, 85.77%, and 62.67%, respectively, all significantly superior to the water control group. The 1:1 combination maintained high efficacy (85.77%) even when the ethylicin dosage was reduced by 50%, and its performance was not significantly different from the single-agent treatment, whereas the 1:3 combination showed significantly lower efficacy (62.67%), indicating an optimal ratio for synergy. Confirming that rhamnolipids can enhance the utilization efficiency of ethylicin through synergistic effects.

### 3.5. Analysis of the Mechanism of Action of Ethylicin

#### 3.5.1. SEM Analysis of Ethylicin-Treated Hyphae

**Figure 8:**
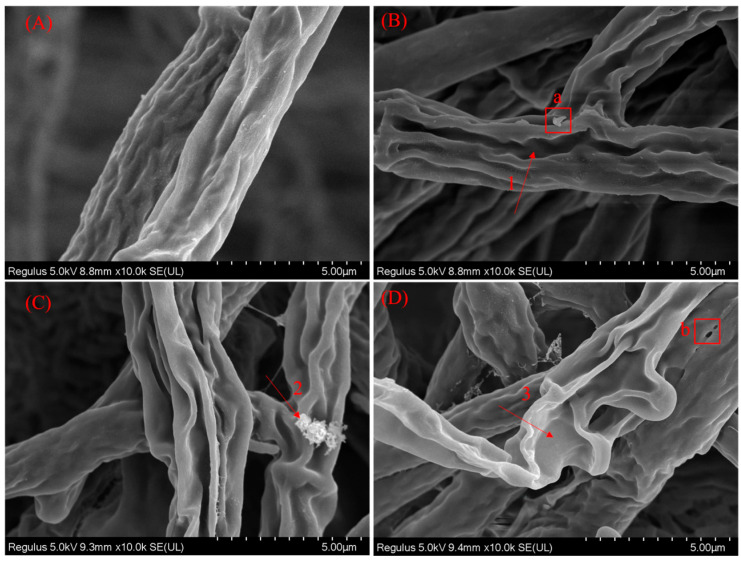
SEM images of the tested fungi treated with ethylicin. Where (**A**): untreated control; (**B**): low concentration (0.21 µg/mL); (**C**): medium concentration (1.75 µg/mL); (**D**): high concentration (7.03 µg/mL).

Scanning electron microscopy (SEM) observations of *F. equiseti* G hyphae revealed (Figure 8) that the treatment with ethylicin induced concentration-dependent damage to the surface morphology of the hyphae. As shown in the figure, (A) untreated control hyphae exhibiting a typical cylindrical structure with fine wrinkles on the surface and an overall intact morphology. (B) Following treatment with a low concentration of ethylicin, deep and regular longitudinal grooves (Arrow 1) appeared along the long axis of the hyphae, with granular substances adherent to localized areas (Box a). (C) After increasing the concentration treatment, the longitudinal grooves became more pronounced and the amount of adhered particles increased (arrow 2). (D) High-concentration treatment resulted in severe morphological alterations, including extensive shriveling of the hyphae, the formation of holes on the surface (Box b), localized contraction, overall flattening, and the complete disruption of the cylindrical structure (Arrow 3).

#### 3.5.2. TEM Analysis of Ethylicin-Induced Ultrastructural Changes

**Figure 9:**
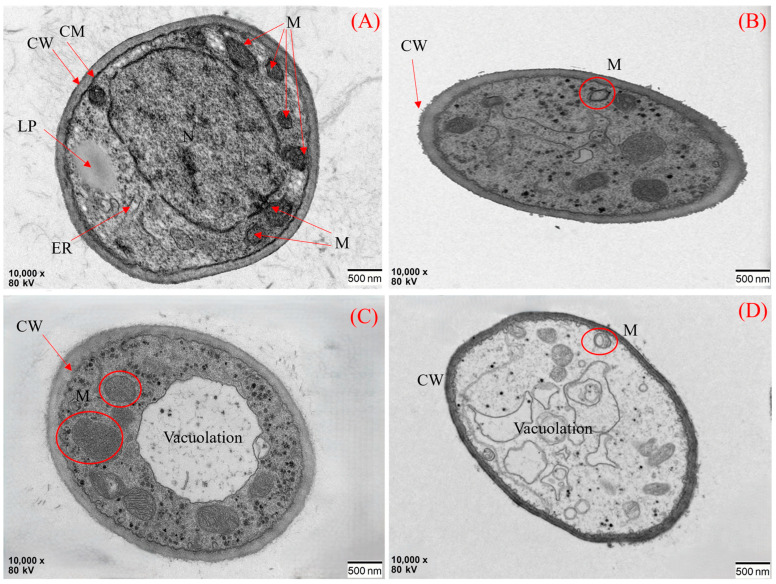
TEM image of ethylicin on the test fungi. Where (**A**): untreated control; (**B**): low concentration (0.21 µg/mL); (**C**): medium concentration (1.75 µg/mL); (**D**): high concentration (7.03 µg/mL). Abbreviations: CM: Cell membrane; M: Mitochondrion; CW: Cell wall; N: Nucleus; LP: Lipid droplet; ER: Endoplasmic reticulum.

Transmission electron microscopy (TEM) analysis of *F. equiseti* G hyphae revealed (Figure 9) that with increasing concentrations of ethylicin (Groups A−D), damage to the fungal cell ultrastructure progressively increased. As shown in the figure, the mycelial surface in Group A was smooth, with an intact cell wall structure, the cell membrane closely adhering to the cell wall, oval-shaped mitochondria with dense cristae, parallelly arranged rough endoplasmic reticulum, lipid droplets distributed within the cell, and a regular-shaped nucleus with an intact nuclear membrane. Group B exhibited initial damage: the cell wall surface became rough, some mitochondrial cristae structures disintegrated, lipid droplets dissolved and disappeared, and the nuclear membrane ruptured, leading to nucleoplasm leakage. In Group C, the damage intensified: vacuolation appeared in the cells, the nucleus was absent, and some mitochondrial cristae structures disintegrated. Group D presented terminal pathological changes: except for some abnormal mitochondria, other organelles had vacuolated, the cell membrane disintegrated, resulting in the spillage of cytoplasmic contents.

## 4. Discussion

This study confirms that *F. equiseti* is the pathogenic fungus causing the gummosis of *Z. bungeanum* on the Qinghai Plateau, and it differs from the strains found in Hanyuan, Sichuan and Longnan, Gansu, indicating geographical adaptive differentiation. Studies have shown that this fungus grows optimally at 22–30 °C and pH 7.2–7.8, which is highly compatible with the average summer temperature (18–25 °C) and rhizosphere soil pH (7.5–8.2) in Qinghai, suggesting that environmental adaptability is a key factor in its emergence as a dominant pathogen [24,25]. High-throughput sequencing further revealed that the relative abundance of the *F. equiseti* is widespread in gummosis-affected soils, whereas this fungus was not detected in healthy soils, which instead exhibited a higher abundance of Mortierellomycota. This is consistent with some studies indicating that *Fusarium* spp. can transmit through soil to cause crop diseases and has the ability to infect plant roots and form colonies within them [26,27,28,29].

This experiment determined through indoor virulence screening that ethylicin exhibits the best inhibitory effect against *F. equiseti*, with an EC_50_ value of 0.396 μg/mL. Its excellent performance makes it a key fungicide for further field trials in fungicide screening. Our electron microscopy analysis directly revealed two key pathological alterations in ethylicin-treated hyphae: (1) rupture of the plasma membrane causing cytoplasmic leakage, (2) disintegration of mitochondrial cristae. This type of structural damage suggests the potential existence of multi-target effects, which is consistent with the previously proposed mechanisms that ethylicin can disrupt cell membrane structure and inhibit biofilm formation [30].

Field efficacy trials have demonstrated that the control efficacy of ethylicin single-agent treatment reaches 93.10%. This agent has already exhibited excellent pharmacokinetic properties in the field: rapid absorption through the phloem (with an absorption rate of >90% within 6 h) and targeted delivery are achieved, while meeting the low residue standards of green pesticides (with a degradation rate of >95% in 5 days) [31]. In this study, the 1:1 formulation of ethylicin–biosurfactant (T2 group) maintained an 85.77% control efficacy while reducing the active ingredient dosage by 50%, indicating that the micellar structure formed by biosurfactant molecules may enhance penetration or delay release, as supported by synergistic mechanisms [32].

This study innovatively employs the Xuan paper imprint method to overcome the quantification challenges of xylem diseases through the principle of physical transfer. By utilizing the dense fiber structure and controllable water absorption of semi-ripe Xuan paper, it achieves clear and reproducible transfers of lesion contours. It provides a semi-quantitative approach for lesion area measurement by integrating ImageJ digital analysis technology and establishes a 0–V grading system to achieve digital expression of disease severity. Compared to traditional visual grading methods, this reduces subjective judgment variability. In contrast to complex techniques such as spectral imaging, its advantages of simple operation and low cost make it well-suited for field operations in plateau environments. The Xuan paper imprint method provides a practical approach for quantitative assessment of gummosis lesions in *Z. bungeanum*. This study supports ethylicin as a key component for the management of gummosis disease on the Qinghai Plateau. Future research should employ omics technologies to elucidate the precise molecular targets of ethylicin’s potential multi-site activity, addressing the limitations of current ultrastructural observations. Additionally, systematic screening of other categories of fungicides should be conducted to expand the chemical arsenal.

## 5. Conclusions

This study identified the primary pathogenic fungus causing the gummosis in Qinghai *Z. bungeanum*, screened and obtained the highly effective fungicidal agent ethylicin (EC_50_ = 0.396 μg/mL), and elucidated its fungicidal mechanism, which involves disrupting the cell membrane structure and inducing mitochondrial damage, with potential multi-target effects suggested by ultrastructural observations and previous studies. The 1:1 combination of ethylicin and rhamnolipids significantly enhances pesticide utilization efficiency, achieving a control efficacy of 85.77%, thereby providing a feasible strategy to reduce chemical pesticide usage. Transmission electron microscopy revealed the molecular mechanism by which ethylicin inhibits fungi through the destruction of fungal ultrastructure, while the Xuan paper imprint method was shown to be a reliable and practical method for field disease spot quantification in this study. To further validate its external validity, future work will establish its correlation with conventional disease assessment methods. This study demonstrates the high efficacy of ethylicin against *Z. bungeanum* gummosis on the Qinghai Plateau under our experimental conditions. The 1:1 combination formulation with reduced ethylicin dosage provides a scientific basis for developing integrated management strategies. Additionally, the novel Xuan paper imprint method offers a practical approach for field quantification of gummosis lesions, though its applicability across diverse hosts and environments requires further validation.

## Figures and Tables

**Table 1:** PCR amplification primer sequences.

Primer Name	Primer Sequence
*ITS*	ITS1: TCCGTAGGTGAACCTGCGG ITS4: TCCTCCGCTTATTGATATGC
*TEF–1α*	TEF1: ATGGGTAAGGARGACAAGAC TEF2: GGARGTACCAGTSATCATGTT
*LSU rRNA*	LROR: ACCCGCTGAACTTAAGC LR5: TCCTGAGGGAAACTTCG
*β–tubulin*	Bt2a: GGTAACCAAATCGGTGCTGCTTTC Bt2b: ACCCTCAGTGTAGTGACCCTTGGC

**Table 2:** Identification information of fungal strains isolated from diseased parts of *Z. bungeanum* trees.

Name	Primer	Accession Number	Primer	Accession Number
A–*Trichoderma longibrachiatum*	*ITS*	PQ498891	*TEF–1α*	PV698514
B–*Penicillium polonicum*	*ITS*	PQ498892	*β–tubulin*	PV698515
C–*Fusarium solani*	*ITS*	PQ498893	*TEF–1α*	PV698516
D–*Mortierella alpina*	*ITS*	PQ498894	*LSU rRNA*	PV667653
E–*Fusarium equiseti*	*ITS*	PQ498897	*TEF–1α*	PV698517
F–*Fusarium oxysporum*	*ITS*	PQ498901	*TEF–1α*	PV698518
G–*Fusarium equiseti*	*ITS*	PQ498902	*LSU rRNA*	PV667654

**Table 3:** Concentration gradients of fungicides tested against *F. equiseti*.

Fungicides	CK	1	2	3	4	5	Sample
90% Carbendazim (WG, Anhui Guangxin Agro-chemical, Xuancheng, Anhui, China)	0	150	164	180	200	225	A
80% Ethylicin (EC, Jiangxi Zhenong Chemi-cal, Nanchang, Jiangxi, China)	0	40	80	160	320	640	B
1.26% Xinjunan Acetate (AS, Shanxi Haozhida Bio-technology, Taiyuan, Shanxi, China)	0	12.6	15.8	21	31.5	63	C
30% Pyraclostrobin (SC, Henan Yongguan Qiaodi Agriculture, Zhengzhou, Henan, China)	0	150	300	600	1200	2400	D
400.0 g/L Flusilazole (EC, Jiangmen Daguangming Agrochemical, Jiangmen, Guangdong, China)	0	20	40	80	160	320	E
98% Rhamnolipid (AS, Ginyung Glycolipid In-dustry (Xi’an) Co., Ltd., Xi’an, Shaanxi, China)	0	4450	5440	7000	9800	16,300	F
350.0 g/L Metalaxyl (ES, Syngenta Crop Protection, Basel, Switzerland)	0	16.7	20	25	33	50	G
98% Actinomycetes (AS, Ginyung Glycolipid In-dustry (Xi’an) Co., Ltd., Xi’an, Shaanxi, China)	0	613	817	1090	1630	3270	H
15% Cupric–Amminium Complexion (AS, Tianjin Green Chemical, Tianjin, China)	0	682	833	1071	1500	2500	I
250.0 g/L Propiconazole (EC, Shandong Weifang Shuangxing Pesticide, Weifang, Shandong, Chi-na)	0	45	50	56	62.5	71	J
20% Triadimefon (EC, Jiangsu Jianpai Agro-chemical, Yancheng, Jiangsu, China)	0	32.5	65	130	260	520	K
40% Dimethomorph (SC, BASF Agricultural Solu-tions, Ludwigshafen, Germany)	0	62.5	125	250	500	1000	L
0.3% Tetramycin (AS, Liaoning Viken Bioengi-neering, Chaoyang, Liao-ning, China)	0	18.75	37.5	75	150	300	M

Where in the table, CK represents the fungal plate without any fungicide added. Since the tests were conducted during the same period, all CKs are identical.

**Table 4:** Toxicity regression equation of fungicides to test strains.

Fungicides	Toxicity Regression Equation	EC_50_ (μg/mL)	95%CI (μg/mL)	Correlation Coefficient (r)
A	*y* = 0.549*x* + 10.4960	0.447 ± 0.045	0.335–0.559	0.9845
B	*y* = 0.671*x* + 6.6651	0.396 ± 0.124	0.086–0.706	0.8797
C	*y* = 0.517*x* + 8.9827	0.451 ± 0.135	0.115–0.787	0.9536
D	*y* = 0.339*x* + 4.6734	1.686 ± 0.253	1.058–2.314	0.9618
E	*y* = 0.144*x* + 6.2528	1.748 ± 0.262	1.098–2.398	0.9546
F	*y* = 0.516*x* + 6.3429	74.087 ± 22.226	18.867–129.307	0.9619
G	*y* =1.431*x* + 14.088	1.748 ± 0.524	0.448–3.048	0.8897
H	*y* = 0.392*x* + 6.1039	59.712 ± 17.914	15.206–104.218	0.8876
I	*y* = 0.660*x* + 8.3445	6.313 ± 1.894	1.606–11.020	0.8029
J	*y* = 0.491*x* + 8.5104	0.781 ± 0.234	0.200–1.362	0.9529
K	*y* = 0.278*x* + 5.7572	0.659 ± 0.198	0.168–1.150	0.8878
L	*y* =0. 154*x* + 4.9402	1.040 ± 0.156	0.653–1.427	0.9459
M	*y* = 0.661*x* + 7.0298	0.464 ± 0.078	0.270–0.658	0.9686

**Table 5:** Methodology evaluation of Xuan paper imprint method.

Sample	Lesion Area (cm^2^)	Paper Area (cm^2^)	CV (%)
1	29.87 ± 4.85	143.48	16.23
2	24.92 ± 2.85	119.36	11.45
3	11.66 ± 2.28	65.46	19.54
4	27.02 ± 3.81	113.75	14.11
5	20.49 ± 3.52	117.00	17.19
6	20.86 ± 2.08	232.50	10.03
7	67.67 ± 1.16	286.75	1.69
8	60.87 ± 7.84	288.00	12.87
9	53.51 ± 6.45	153.00	12.04
10	21.58 ± 3.09	91.65	14.31

**Table 6:** Control effect of different concentrations of ethylicin.

Group	0	I	II	III	IV	DI (%)	Control Efficacy (%)
Water	0	12.00 ± 2.94	16.00 ± 3.27	15.00 ± 2.16	7.00 ± 1.41	46.80 ± 1.50	–
1:0	45.00 ± 2.16	2.67 ± 1.70	2.00 ± 0.82	0.33 ± 0.47	0	3.23 ± 0.10	93.10 ± 1.90 a
1:1	38.00 ± 2.16	7.67 ± 1.70	4.00 ± 0.82	0.33 ± 0.47	0	6.66 ± 0.10	85.77 ± 2.38 a
1:3	20.00 ± 1.63	21.33 ± 1.25	3.67 ± 1.25	5.00 ± 1.63	0	17.47 ± 2.45	62.67 ± 4.09 b

Where different letters indicate significant differences (*p* < 0.05, one-way ANOVA with Tukey’s test).

## Data Availability

The original contributions presented in this study are included in the article. Further inquiries can be directed to the corresponding authors.
